# Prevalence of *Escherichia coli* and Antibiotic-Resistant Bacteria During Fresh Produce Production (Romaine Lettuce) Using Municipal Wastewater Effluents

**DOI:** 10.3389/fmicb.2021.660047

**Published:** 2021-05-20

**Authors:** Harvey N. Summerlin, Cícero C. Pola, Eric S. McLamore, Terry Gentry, Raghupathy Karthikeyan, Carmen L. Gomes

**Affiliations:** ^1^Department of Biological and Agricultural Engineering, Texas A&M University, College Station, TX, United States; ^2^Department of Mechanical Engineering, Iowa State University, Ames, IA, United States; ^3^Department of Agricultural Sciences, Clemson University, Clemson, SC, United States; ^4^Department of Soil and Crop Sciences, Texas A&M University, College Station, TX, United States

**Keywords:** wastewater, produce irrigation, water reuse, fecal coliforms prevalence, antibiotic-resistant bacteria, food safety

## Abstract

High demand for food and water encourages the exploration of new water reuse programs, including treated municipal wastewater usage. However, these sources could contain high contaminant levels posing risks to public health. The objective of this study was to grow and irrigate a leafy green (romaine lettuce) with treated wastewater from a municipal wastewater treatment plant to track *Escherichia coli* and antibiotic-resistant microorganisms through cultivation and post-harvest storage to assess their fate and prevalence. Contamination levels found in the foliage, leachate, and soil were directly (*p* < 0.05) related to *E. coli* concentrations in the irrigation water. Wastewater concentrations from 177 to 423 CFU ml^−1^ resulted in 15–25% retention in the foliage. Leachate and soil presented means of 231 and 116% retention, respectively. *E. coli* accumulation on the foliage was observed (*p* < 0.05) and increased by over 400% during 14-day storage (4°C). From randomly selected *E. coli* colonies, in all four biomass types, 81 and 34% showed resistance to ampicillin and cephalothin, respectively. Reclaimed wastewater usage for leafy greens cultivation could pose potential health risks, especially considering the bacteria found have a high probability of being antibiotic resistance. Successful reuse of wastewater in agriculture will depend on appropriate mitigation and management strategies to guarantee an inexpensive, efficient, and safe water supply.

## Introduction

As world population and demand for food increase, safe water for agricultural use has become increasingly scarce. The water footprint of humanity is estimated at 9,087 km^3^ year^−1^, of which agriculture accounts for 92% (Hoekstra and Mekonnen, 2012). In some areas, surface water is not readily available, and other options, such as drilling a well, are not cost-effective. Non-traditional water supplies, such as treated municipal wastewater for irrigation, have the potential to meet increasing water demands and conserve current potable supplies; however, wastewaters often have microbial and chemical contaminants that may affect public health and/or environmental quality. Wastewater treatment (WWT) strategies and advanced irrigation systems may limit exposure of crops, animals, humans, and groundwater to contaminants. Several irrigation practices, such as drip, flood, and subsurface irrigation techniques used with treated wastewater, have been reported to mitigate the risk of contamination (Solomon et al., 2002; Pavione et al., 2013). However, due to the morphology of certain plants, such as lettuce or spinach, commercial-scale production requires canopy (or spray) irrigation. This irrigation process involves water coming into direct contact with the edible foliage, which poses a higher risk of contamination (Robinson, 2002).

A review by De Keuckelaere et al. (2015) reports that there are few site-specific data points available for risk assessment related to use of water and food safety of fresh produce. Specific parameters lacking hard data include rate of pathogen transfer from irrigation water to crops, and pathogen fate, transport, and survival in or on food crops. Furthermore, precise information regarding fecal coliforms, pathogens, and antibiotic-resistant bacteria (ARB) accumulation during and after harvest and their potential effect on future crops along with risks posed to human health are scarce. Therefore, in order to create adequate risk management practices and guidelines for wastewater irrigation in agriculture, site-specific studies of bacteria motility and accumulation are critical, as it is becoming an increasingly popular alternative.

Nowadays, consumption of fresh produce is on the rise, due to its associated health and nutritional benefits. At the same time, fresh produce is one of the leading causes of foodborne illnesses (Rai and Tripathi, 2007) with 377 outbreaks reported by the United States Centers for Disease Control and Prevention (CDC) from 2004 to 2012 (Callejon et al., 2015). Foodborne illnesses can emerge from poor water quality used during fresh produce production. For instance, a multistate outbreak of *E. coli* O157:H7 infections linked to romaine lettuce that infected 210 people from 36 states and caused five deaths indicated that the source of *E. coli* O157:H7 was likely from the canal water (Yuma growing region) used to irrigate the romaine lettuce (CDC, 2018). Moreover, the overuse of antibiotics can be directly related to the occurrence and propagation of ARB, which have been increasing rapidly over the past decades (Edberg et al., 2000; WHO, 2006; Bitton, 2010). The ARB issue has been identified by many global public health entities including the World Health Organization and CDC as a critical concern (Bitsch et al., 2014).

Several studies have examined the effects and risks of using wastewater effluents to irrigate fresh produce such as lettuce, spinach, rocket, and tomato (Assadian et al., 2005; Ribera and McCorkle, 2012; De Keuckelaere et al., 2015). Throughout these studies, multiple factors were tested to observe their effects on the prevalence of fecal indicator bacteria (FIB), which were used to estimate the levels of harmful pathogens for risk assessment (Mena and Pillai, 2008; Alam et al., 2014). Additionally, these previous studies reported on the levels of crop contamination; however, these studies did not show how the entire system of foliage, soil, and leachate is affected over time when using wastewater irrigation.

Reclaimed wastewater in agriculture has the potential to provide alternative irrigation and nutrient sources in water-scarce regions and consequently promote water conservation. However, to develop mitigation plans to protect public health, site-specific evaluation of bacteria movement and persistence is needed. In this study, lettuce was irrigated with secondary treated wastewater to track the fate and prevalence of *E. coli* and ARB throughout the entire system (foliage, soil, and leachate) during cultivation and post-harvest storage.

## Materials and Methods

### *Escherichia coli* Monitoring in Fresh Produce Materials

#### Wastewater

Wastewater was obtained weekly from the Texas A&M WWT Plant, College Station, TX, United States. The wastewater was collected after solids removal and secondary clarification processes. Three liters were collected using a beaker affixed to a pole and placed into sterile plastic jugs for transport to the laboratory. A sample (10 ml) of the wastewater was reserved for further analysis (described below).

#### Leafy Greens

Twelve young 15-cm romaine lettuce plants (*Lactuca sativa* var. longifolia, Bonnie Plants, Union Springs, AL, United States) were purchased from a local nursery. The plants were placed into 20-cm diameter plastic pots and filled with EcoScraps moisture retaining potting soil (EcoScraps Co., South Jordan, UT), leaving a 2-cm lip to the top. The potting soil was sterilized in an autoclave for 90 min at 121°C and analyzed by the Texas A&M Department of Soil and Crop Sciences Laboratory (College Station, TX) generating the following results: pH: 7.2, nitrate: 0 ppm, phosphorus: 95 ppm, potassium: 441 ppm, moisture content: 4.44%, soil composition with sand: 91.2%, clay: 2.6%, silt: 6.1%, and total solids: 55.63%. A suggested supplement of nitrogen was applied in the amount of 0.68 g cm^−2^.

Lettuce plants were transplanted and grown using sterile reverse osmosis (RO) water for 14 days prior to the irrigation experiment. Each row of six plants was grown under two 2-Light T12 fluorescent shop lights (Lithonia Lighting, Conyers, GA) containing four 1.22 m 40-watt fluorescent tube light bulbs (General Electric, Fairfield, CT). The bulbs provided 2,900 lumens each and consisted of two 6,500 K and two 3,000 K color temperature bulbs to resemble natural daylight. The lighting fixtures were plugged into a wall outlet timer that allowed 14 h of continuous light located 15 cm above the plants. Temperature (23 ± 2 C) and relative humidity (55 ± 4%) were kept constant throughout the experiment.

#### Inoculation

**Figure 1:**
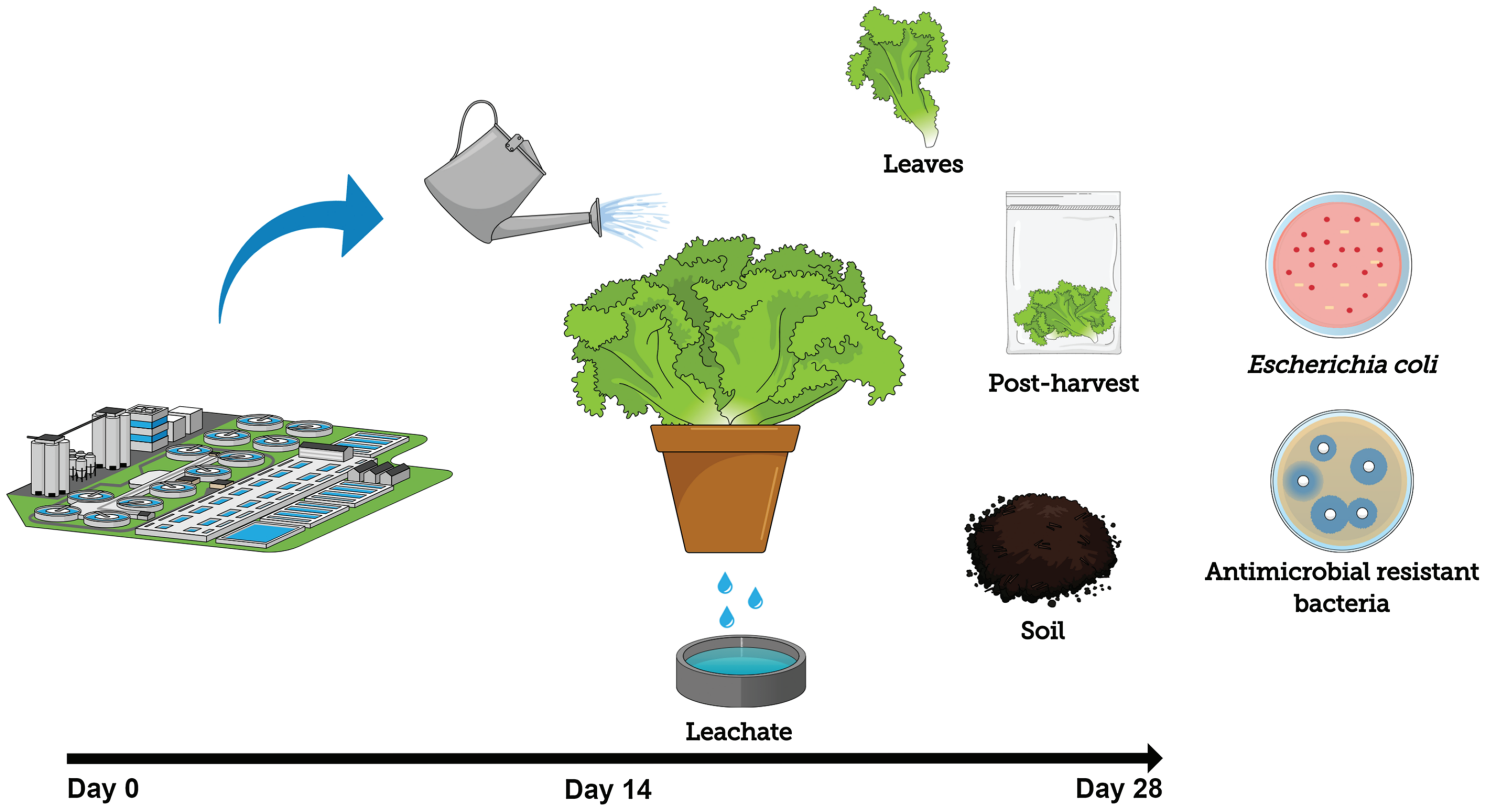
Experimental design schematic showing lettuce cultivation using secondary-treated wastewater as irrigation source to track the fate and prevalence of *Escherichia coli* and antibiotic-resistant bacteria (ARB) throughout the entire system (foliage, soil, and leachate) during cultivation (14-day period) and post-harvest storage at 4°C (14-day period).

Once a week, for 3 weeks, approximately 15 ml of wastewater was applied directly to each plant’s foliage using a 150-ml sterile spray bottle (Apothecary Products, Inc., Minneapolis, MN) with the nozzle on mist position, thoroughly covering each side of all leaves from a 15-cm distance. Then, 150 ml of the wastewater was poured into each plant’s pot, completely soaking the soil following good agricultural practice recommendations. These irrigation methods were applied to mimic sprinkler and furrow irrigation, which are typically used for leafy greens cultivation (Masabni et al., 2009). Plants were also supplemented with 50 ml of RO water each day for the rest of the week to prevent drying out and wilting. The experimental design is summarized in Figure 1. All procedures were performed inside a biosafety cabinet.

#### Sample Collection and Analysis

A 10-ml sample of the wastewater was taken and placed into a sterile conical centrifuge tube (VWR International, Radnor, PA). After wastewater irrigation, 10 ml of leachate from each plant was immediately collected from the pot saucer. Foliage samples were collected 1 h after irrigation, by cutting the outermost leaves from their stems. Leaf blades were removed from the vein and cut into 2.5-cm strips. From each plant, 5 g of foliage was stored into sterile Whirl-Pak^®^ bags (eNasco, Fort Atkinson, WI). Then, 10 ml of buffered peptone water (BPW, Becton, Dickinson and Company, Franklin Lakes, NJ, United States) was added to each foliage sample to create a 1:2 ratio of foliage to buffer suspension, and the bags were homogenized for 2 min. Post-harvest foliage samples were collected at the same time as the pre-harvest day 14 samples and stored at 4°C in sterile plastic bags (low-density polyethylene, Whirl-Pak^®^ bags) for 7–14 days when BPW was then added and samples were processed accordingly. Dry weight for foliage samples was obtained by moisture content measurement following AOAC method 930.04 (AOAC, 1990).

Soil samples were collected 6 h after irrigation to allow adequate drainage. From each pot, a sterile 2-cm-diameter core tube was inserted 5 cm deep to collect 2 g of soil. Then, 8 ml of BPW was added to create a 1:4 ratio of soil to buffer suspension. The collected samples were then vortexed for 30 s to homogenize the contents. Subsequently, these samples were allowed to settle for 10 min to separate the soil from buffer.

Aliquots (0.1 ml) of the samples were then plated on MacConkey Agar (Hardy Diagnostics, Santa Maria, CA) using the spread plating method. Several serial dilutions of all samples were plated to ensure samples were within the limits of detection. Plates were incubated overnight at 35°C. Two plates per dilution of each sample were plated, counted, and reported in either CFU g^−1^ or CFU ml^−1^ of sample (USFDA, 1998).

#### Antibiotic-Resistant Bacteria

Ten *E. coli* colonies from each of the four materials collected (i.e., wastewater, soil, leachate, and foliage) for a total of 40 *E. coli* isolates were randomly selected per pre-harvest sampling time, and similarly, 10 *E. coli* colonies from foliage per post-harvest sampling time. Individual colonies were collected, suspended in BPW, streaked on Tryptic soy agar (TSA, Becton, Dickinson and Company, Franklin Lakes, NJ, United States), and incubated overnight at 35°C in accordance with the Kirby-Bauer method (USFDA, 1998). Next, *E. coli* cell suspensions were then prepared by placing two isolates into tubes containing 5 ml of Tryptic soy broth (TSB, Becton, Dickinson and Company, Franklin Lakes, NJ, United States) and incubating at 35°C for 3 h while shaking at 150 rpm in a water bath (VWR International). Tubes were checked for appropriate turbidity with 0.5 McFarland standard, which corresponds to a 10^8^ CFU ml^−1^ bacterial cell count (USFDA, 1998).

*Escherichia coli* suspensions were then re-streaked onto Muller Hinton Agar (MHA, Becton, Dickinson and Company, Franklin Lakes, NJ, United States) plates. Then, antibiotic resistance of the colonies was determined by the Kirby-Bauer method for antibiotic susceptibility. Eight antibiotic susceptibility disks (Becton, Dickinson and Company, Franklin Lakes, NJ) of ampicillin (10 μg), cefoperazone (75 μg), cephalothin (30 μg), ciprofloxacin (5 μg), gentamicin (120 μg), imipenem (10 μg), sulfamethoxazole/trimethoprim (23.75/1.25 μg), and tetracycline (30 μg) were stamped onto each MHA plate using a BBL^®^ Sensi-Disc^®^ 8-place Dispenser (Becton, Dickinson and Company, Franklin Lakes, NJ, United States). The stamped MHA plates were incubated for 24 h at 35°C. Then, the zones of inhibition (ZOI) were measured to determine resistance, intermediate resistance, or susceptibility to each antibiotic, according to the Clinical and Laboratory Standards Institute standards (Bauer et al., 1966).

#### Data Analysis

All experiments were performed in triplicate as independent experiments, and the results are expressed as mean ± standard deviation. Differences among variables were tested using one-way ANOVA with a significance level of 5%, and significantly, different means were separated by the Tukey HSD test. All data were analyzed using JMP^®^Pro statistical software (SAS, Cary, NC 27513). Due to the rain event prior to day 14, large input (wastewater) variability of *E. coli* contamination levels in foliage, soil, and leachate was normalized by transforming CFU ml^−1^ to percentage of *E. coli* retention. Response material (i.e., soil, leachate, and foliage) *E. coli* concentration (CFU ml^−1^) was divided by wastewater *E. coli* concentration (CFU ml^−1^) to yield retention as a percentage. Beginning with pre-harvest data, statistical analysis was performed using a Tukey HSD *post-hoc* test to compare *E. coli* prevalence over time among all materials (wastewater, leachate, foliage, and soil). Then, differences in means were analyzed among materials for each sampling time. Finally, post-harvest foliage samples were analyzed to detect mean differences over storage time.

Antibiotic-resistant bacteria samples were analyzed, with focus on the three most common resistance patterns observed from ARB analysis: ampicillin (10 μg), cephalothin (30 μg), and ciprofloxacin (5 μg). In this study, intermediate resistant and resistant bacteria were combined and expressed as “resistant” to simplify the results. Resistance among samples was expressed as a percentage of each sampling population. ARB results were compared over time among all materials. Then, mean differences were analyzed among materials for each sampling time. All analyses were performed by using Tukey HSD *post-hoc* test to separate differences in means and Levene’s test to test for homoscedasticity.

## Results and Discussion

### Fresh Produce Materials and *E. coli* Monitoring

The concentrations of *E. coli* present in the irrigation water varied and were recorded in log CFU ml^−1^ as 2.3 ± 0.0, 2.6 ± 0.2, and 5.1 ± 0.1 log CFU ml^−1^ for days 0, 7, and 14, respectively. There was a large rain event of 32.8 mm (Weather Underground, 2016) in the Bryan/College Station (Texas) area on November 6, 2016, 1 day prior to the collection of the day 14 sample. It was also observed that the WWT plant was not operating at full effectiveness because of a failure in the aeration system, which provides oxygen to microorganisms in the solids removal tank. The WWT plant reported an *E. coli* concentration of 2.5 ± 0.6 log CFU 100 ml^−1^ in UV-treated effluent on day 14 and an average of 2.6 ± 2.0 log CFU 100 ml^−1^ in the 4 days following the rain event and system failure. These are significantly higher concentrations than the average for the rest of the month which was 1.0 ± 0.7 log CFU 100 ml^−1^, which consequently affected the results by introducing very large concentrations of *E. coli* that were significantly different (*p* < 0.05) than the previous two irrigations. The EPA standard for final effluent discharge is a geometric mean of 2.1 log CFU 100 ml^−1^ (USEPA, 2017).

For irrigation water, including alternative water sources, the *E. coli* concentration must not exceed 126 CFU 100 ml^−1^ (geometric mean or 2.10 log CFU 100 ml^−1^) without triggering a responsive action (FDA, 2017; EC, 2020; EPA, 2021). This study was carried out with an initial *E. coli* concentration that exceeded regulatory requirements in order to track the fate and prevalence of *E. coli* and ARB throughout the entire system (foliage, soil, and leachate) during cultivation and post-harvest storage, and also to demonstrate the worst-case scenario in the event of high *E. coli* levels in irrigation water. Recent reviews have addressed the technological challenges of implementing these federal guidelines for the specific case of alternative water sources (e.g., treated wastewater and brackish water; Markland et al., 2017; Rock et al., 2019) that would benefit with data-informed decision support tools being actively used to monitor microbial contamination (McLamore et al., 2019; Giacobassi et al., 2021).

A recent study conducted by Solaiman et al. (2020) assessed the prevalence of bacteria indicating water quality, fecal contamination and crop contamination risk (*E. coli*, total coliforms, *Enterococcus*, and *Aeromonas*) over a 26-month longitudinal study in the mid-Atlantic region of the United States. For all water types, higher *E. coli* counts (*p* < 0.05) were observed in the vegetable crop growing (May-October) than non-growing (November-April) season. Additionally, this study found that bacterial counts in reclaimed water generally met microbial standards by federal guidelines or needed minimal mitigation (Solaiman et al., 2020). Another recent work studied the prevalence of Shigatoxigenic *E. coli* (STEC) and atypical enteropathogenic *E. coli* (aEPEC) in untreated surface water and reclaimed water in the mid-Atlantic United States (Haymaker et al., 2019). These pathogenic strains were selected since they have been responsible for several outbreaks of infections associated with leafy greens consumption recently. The study found that 2.35% (12/510) of water samples contained STEC isolates, while 9.0% (46/510) contained aEPEC isolate. The authors pose that STEC isolates at reclaimed water sites may have been introduced after WWTs (Haymaker et al., 2019), which reinforces the need to monitor irrigation water quality to minimize the risk of foodborne illnesses associated with leafy greens.

**Figure 2:**
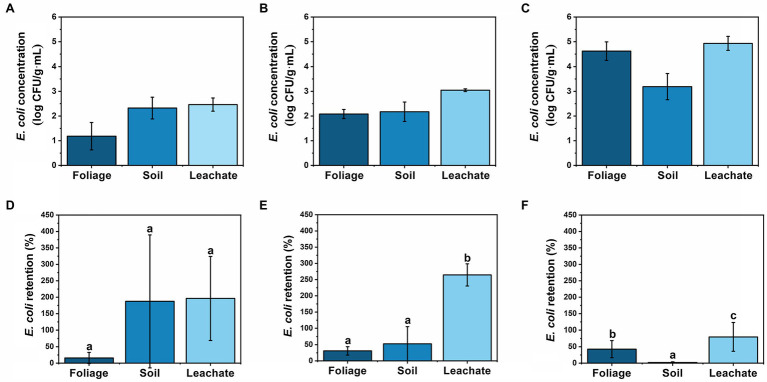
Comparison of *E. coli* concentration at **(A)** day 0, **(B)** day 7, and **(C)** day 14 in log CFU g^−1^ or log CFU ml^−1^ (wet weight basis) and retention on **(D)** day 0, **(E)** day 7, and **(F)** day 14 in % for foliage, soil, and leachate samples, respectively. Retention was calculated by dividing *E. coli* sample concentration (CFU ml^−1^ or CFU g^−1^) by weekly *E. coli* irrigation water concentration (CFU ml^−1^). Moisture content of foliage = 91.3% and soil = 4.44%. Sample sizes: wastewater = 10 ml, leachate = 10 ml, foliage = 5 g, and soil = 2 g. Error bars denote standard deviation for arithmetic mean (*n* = 6 for day 0 foliage and *n* = 12 for all others). Different letters indicate statistical difference using Tukey-Kramer HSD, *α* = 0.05.

**Figure 3:**
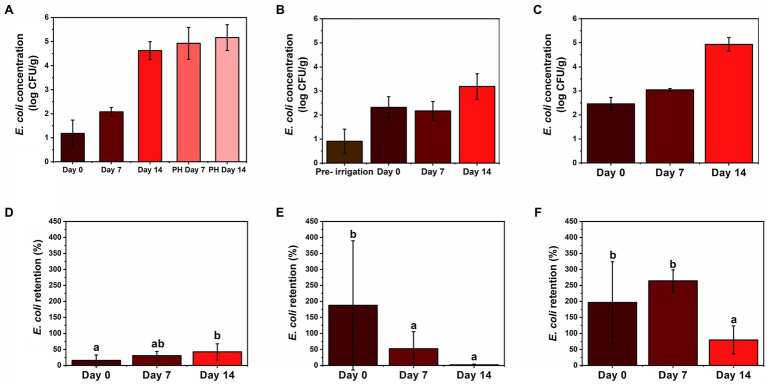
Comparison of *E. coli* concentration on **(A)** foliage, **(B)** soil, and **(C)** leachate in log CFU g^−1^ or log CFU ml^−1^ (wet weight basis) and retention on **(D)** foliage, **(E)** soil, and **(F)** leachate in % over time (days 0, 7, and 14 and post-harvest (PH) days 7 and 14), respectively. Retention was calculated by dividing *E. coli* sample concentration (CFU ml^−1^ or CFU g^−1^) by weekly *E. coli* irrigation water concentration (CFU ml^−1^). Moisture content of foliage = 91.3% and soil = 4.44%. Sample sizes: wastewater = 10 ml, leachate = 10 ml, foliage = 5 g, and soil = 2 g. Error bars denote standard deviation for arithmetic mean (*n* = 6 for day 0 foliage and *n* = 12 for all others). Different letters indicate statistical difference using Tukey-Kramer HSD, *α* = 0.05.

Initially, soil and leachate displayed higher concentrations of *E. coli* than foliage until day 14 when foliage surpassed the soil concentrations (Figures 2A–C). Overall, each material showed an increase in *E. coli* concentration from the previous sampling time except for soil on day 7 (Figure 3B). Foliage consistently increased the concentration throughout cultivation and post-harvest storage (Figure 3A). Meanwhile, leachate samples had the largest concentrations at each sampling time (Figure 3C). Foliage displayed a positive trend in retention with 16, 31, and 43% on days 0, 7, and 14, respectively (Figure 3D). This shows that there was accumulation of *E. coli* on the foliage throughout the cultivation process. The bacteria were able to survive and persist on foliage for more than 1 week. Similar results were observed by Alam et al. (2014), which studied cessation of irrigation prior to harvest, and how the elapsed time affected *E. coli* concentration. In a recent study by Allard et al. (2019), fecal indicators, pathogenic bacteria, and total bacterial communities were tracked from a creek water irrigation source used to irrigate fresh produce *via* drip irrigation to assess the impact of irrigation events on soil and produce microbiota. The study reported that total coliforms in soil were significantly increased immediately and 3 days post-irrigation compared to pre-irrigation, and *E. coli* level in soil increased after irrigation; however, the difference was not significant, and bacterial die-off was not observed neither in soil nor on produce (Allard et al., 2019).

Soil and leachate retention rates were often higher than 100% (Figures 3E,F) which shows that the soil was not completely sterile prior to the first irrigation of wastewater on day 0. Possibly, the soil in the lettuce transplants was contaminated with *E. coli*, which propagated during the first 2 weeks of sterile irrigation prior to day 0. Prior to any wastewater application, there was no detectable *E. coli* on the foliage, and the autoclaved soil had 13 ± 10 CFU g^−1^ of *E. coli*, presenting a 25-fold increase after first irrigation. These findings are similar to Orlofsky et al. (2016), which studied the correlation of FIB and pathogens found on fresh crops irrigated with different types of water, including potable, secondary-treated wastewater (TWW), and tertiary TWW, and found *E. coli* in soil, which had only been irrigated with potable water. Conversely, in the present study, soil concentrations were relatively consistent but displayed a negative trend in retention with 188, 53, and 2% on days 0, 7, and 14, respectively (Figures 3B,E). This result suggests a maximum contamination load in the soil and that the excess of *E. coli* will stay in the irrigation water to become leachate. Such results are particularly important for low-growing crops, since they have a closer contact with the ground, increasing the infection risk coming from the contaminated soil (Pavione et al., 2013). Allard et al. (2019) showed that when using drip irrigation to cultivate kale and radish from creek water as irrigation source, even though target pathogens (*Salmonella enterica* and *Listeria monocytogenes*) were detected in irrigation water, they were not likely transferred to the field *via* drip irrigation (i.e., only one post-irrigation kale sample was positive for *S. enterica*). However, this study reported that elevated total coliforms and *E. coli* levels in surface water irrigation influenced bacterial communities in soil and on produce (Allard et al., 2019).

Leachate exhibited the largest retention rates among response materials during the cultivation process (Figures 2D–F). The leachate collected the existing *E. coli* in the soil in addition to the *E. coli* introduced by the irrigation water, yielding a retention rate greater than 100% for days 0 and 7 (Figure 3F). According to Dwivedi et al. (2016), the saturated water content of the soil is an important parameter in subsurface *E. coli* transport, and in this study, sterile supplemental water was provided during cultivation to avoid drying out and wilting of the lettuce. Similar to soil, day 14 leachate retention was affected by large input concentration and was significantly less than the previous two sampling times, dropping to 80% (Figure 3F), even though accumulation increased over time (Figure 3C). Our results show that contaminated water can penetrate through 15 cm of soil, but further investigation is needed to determine *E. coli’s* fate as water percolates down to groundwater reservoirs. According to Stall et al. (2014), depth of soil has a positive effect on reducing *E. coli* concentrations in leachate.

*Escherichia coli* concentration in foliage increased during post-harvest storage at 4°C (Figure 3A). Similar results were reported by Lopez-Velasco et al. (2010), which studied the effect of post-harvest storage temperatures (4 and 10°C) and times (5, 10, and 15 days) on *E. coli*-contaminated spinach. Even though this is not the ideal temperature for *E. coli* growth (35 ± 2°C), stress response mechanisms can trigger the expression of genes, such as RpoS which is believed to be directly related to the synthesis of internal trehalose in the bacterial cell resulting in the increase of cold resistance (Battesti et al., 2011). *E. coli* counts increased from 4.6 ± 0.4 log CFU g^−1^ on the harvest day to 4.9 ± 0.7 log CFU g^−1^ after 7 days of refrigerated storage. After 14 days of storage, 5.2 ± 0.5 log CFU g^−1^ was observed, a 200% increase from day 7 of post-harvest storage. Days 7 and 14 were significantly different (*p* < 0.05) than day 0, but not significantly different from each other (*p* > 0.05, Figure 3A). These results support the importance of fresh produce being free of any pathogenic microbial contamination during cultivation and processing, as *E. coli* left on the surface can quickly propagate at recommended storage temperature (4°C) and pose health risks to consumers if no disinfection treatments are applied prior consumption (Lopez-Velasco et al., 2010). For post-harvest, there is a requirement of no detectable generic *E. coli* in 100 ml of water used in direct contact with produce or on food contact surfaces (FDA, 2017). Additionally, the United States Food and Drug Administration Food Safety Modernization Act established standards in a Produce Safety Rule specific to pre-harvest agricultural water that will come in direct contact with edible portions of fresh produce crops during cultivation including mitigation measure of allowing up to 4 days elapse between irrigation and harvest to allow for bacterial die-off (Havelaar et al., 2017; FDA, 2020, 2021a). These requirements in combination with the “hold and test” policy adopted in 2012 by the United States Department of Agriculture (USDA) significantly reduce the risk of consumer exposure to unsafe products *via* food recalls (USDA-FSIS, 2013). Notably, to date, there have been no reported foodborne illnesses resulting from the use of reclaimed water (tertiary treated) in irrigation practices in the United States (EPA, 2021; FDA, 2021b).

### Antibiotic-Resistant Bacteria

**Figure 4:**
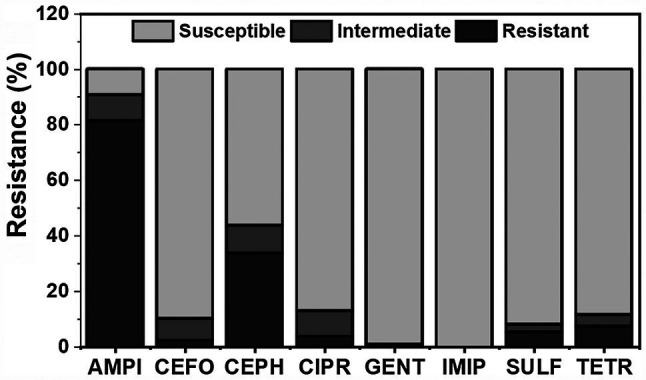
Distribution of antibiotic resistance (resistant, intermediate, and susceptible) among all materials (wastewater, foliage, leachate, and soil) for eight different antibiotics tested (AMPI, ampicillin; CEFO, cefoperazone; CEPH, cephalothin; CIPR, ciprofloxacin; GENT, gentamicin; IMIP, imipenem; SULF, sulfamethoxazole; and TETR, tetracycline) throughout romaine lettuce production (days 0, 7, and 14, post-harvest day 7, and post-harvest day 14). Total *E. coli* isolates were *n* = 140.

A total of 140 *E. coli* isolates across all sampling times and materials were tested for antibiotic resistance against eight antibiotics. Ampicillin had the highest recorded resistance among isolates at 81% (*n* = 114), followed by cephalothin at 34% (*n* = 47; Figure 4). Silva et al. (2006) reported that WWT plants have generally been ineffective at removing certain strains of resistant bacteria, specifically *Enterococcus* isolates resistant to the antibiotics ciprofloxacin, erythromycin, and tetracycline, and that the prevalence of ciprofloxacin resistance increased throughout the treatment process. Recently, Chopyk et al. (2020) characterized the taxonomic and functional variations in microbial communities of untreated surface and reclaimed water used in irrigation applications in the mid-Atlantic region of the United States. Among their findings, antimicrobial resistance genes to commonly used antibiotics (aminoglycosides, sulfonamides, rifamycins, macrolides, cephalosporins, fluoroquinolones, and tetracyclines) were found with the highest diversity and abundance in samples from a reclamation facility and a wastewater-impacted freshwater creek (Chopyk et al., 2020). Additionally, the authors reported that bacterial community characteristics varied depending on the date sampled and the specific site (Chopyk et al., 2020), which corroborates with this study findings. Gentamicin and imipenem displayed the lowest rate of resistance, with 1% (*n* = 1) and 0%, respectively. Several antibiotics including ampicillin (*n* = 13), cefoperazone (*n* = 11), cephalothin (*n* = 14), and ciprofloxacin (*n* = 13) displayed larger intermediate rates of resistance, ranging from 8 to 10% of all isolates. These findings are important because there is a high probability that these organisms will adapt to their environment and become more resistant, as suggested by Lagacé-Wiens et al. (2013). For this reason, isolates displaying intermediate resistance were categorized as resistant for the remainder of analysis, similar to Laird (2016).

**Figure 5:**
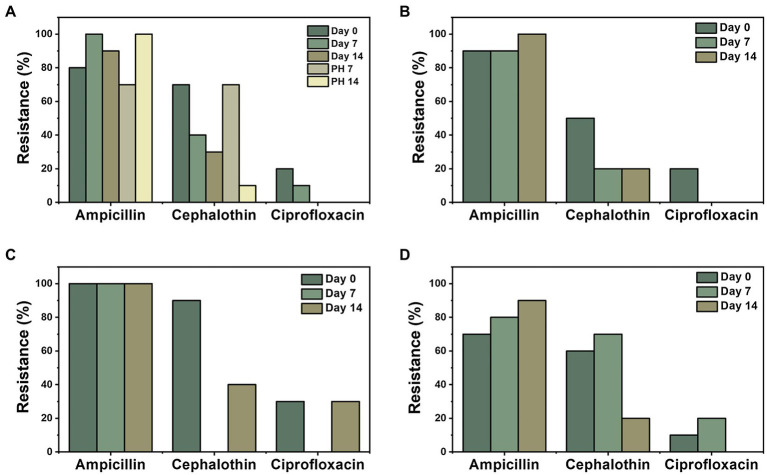
Distribution of ARB over time (days 0, 7, and 14 and post-harvest (PH) days 7 and 14) for three antibiotics that displayed the highest prevalence of resistance (ampicillin, cephalothin, and ciprofloxacin) among response materials: **(A)** foliage, **(B)** leachate, **(C)** soil, and **(D)** wastewater source.

Three antibiotics with the highest combined prevalence of resistance and intermediate resistance were selected to further investigate their fate and transport throughout fresh produce production (Figure 5). These antibiotics were ampicillin, cephalothin, and ciprofloxacin with 90% (*n* = 127), 44% (*n* = 61), and 13% (*n* = 18) rate of resistance in all isolates, respectively. For an *E. coli* isolate to be categorized as resistant or intermediate resistant, the bacterial lawn on the Kirby-Bauer plate had to show little to no ZOI around a given antibiotic disc (Bauer et al., 1966). As shown in Figure 5A, ampicillin had the highest overall resistance prevalence in foliage. There was no distinct trend or significant differences in antibiotic resistance over the duration of the experiment. Of the 20 post-harvest foliage samples, 17 samples (85%) were resistant to ampicillin. Conversely, only 5% (*n* = 1) of post-harvest foliage isolates were resistant to cephalothin and 0% to ciprofloxacin. In the United States, ampicillin and ciprofloxacin are two of the top five antibiotics prescribed to adults (Shapiro et al., 2014). These antibiotics have been found in WWTPs in varying concentrations and treatment plant designs (Batt et al., 2007) due to their frequent use in the past and today’s society, which suggests that treatment plants may be contributing to the prevalence of ARB found downstream.

**Figure 6:**
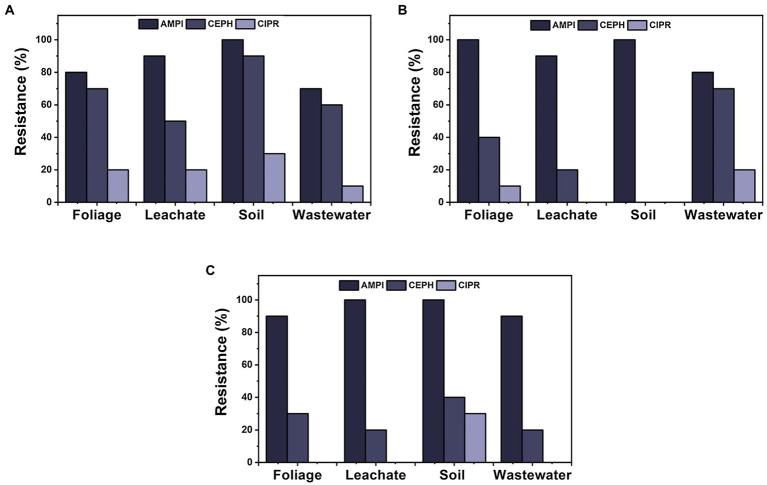
Comparison of ARB for three antibiotics that displayed the highest prevalence of resistance (AMPI, ampicillin; CEPH, cephalothin; and CIPR, ciprofloxacin) among materials over sampling times during cultivation: **(A)** day 0, **(B)** day 7, and **(C)** day 14.

Day 0 sampling time displayed the largest resistance in isolates from ampicillin (*n* = 34), followed by cephalothin (*n* = 27) and ciprofloxacin (*n* = 8), for all four materials tested (Figure 6). Furthermore, ampicillin was the most prevalent isolate resistance among each material throughout sampling times (*n* = 127). Ciprofloxacin showed the least resistance (13%) of the three selected antibiotics, for all materials for each sampling day, except for day 7 for soil samples, where both cephalothin and ciprofloxacin showed 0% isolate resistance. For overall ampicillin resistance, soil and wastewater were significantly different from each other (*p* = 0.049); however, no significant differences were observed from foliage nor leachate. There were no other significant differences among sample materials for cephalothin and ciprofloxacin.

Tracking ARB in leachate and soil in addition to foliage is equally important since these bacteria can make their way back into water sources like rivers and creeks, *via* runoff and leaching. Furthermore, moist soil provides an optimal environment for resistant bacteria to propagate and pass along resistant genes (Orlofsky et al., 2016). Consequently, water systems are a key vehicle for these bacteria containing antibiotic resistant traits to propagate, multiply, and transfer their resistant genes (Pei et al., 2006). Extensive research on the fate and transport of ARB in water sources resulting from livestock production has been carried out (Addison, 1984; Humphrey et al., 2005). A recent study by Malayil et al. (2020) described the metabolically active bacteria diversity and abundance from reclaimed water and agricultural ponds used as alternative irrigation water sources from the mid-Atlantic United States region. The study observed that antimicrobial resistance and virulence gene profiles appeared to be more diverse and abundant in relic (inactive) DNA than in viable cells (metabolically active) in the tested water types with *Actinobacteria*, *Flavobacterium* spp., *Pseudomonas* spp., and *Aeromonas* spp. being the most abundant and metabolic-active microorganisms (Malayil et al., 2020). Our study presented some baseline information on the prevalence of viable ARB during fresh produce production irrigated with treated municipal wastewater. Further studies are needed to identify potential mitigation and intervention points in the farm-to-fork continuum when treated wastewater effluents for irrigation of fresh produce.

This study has shown the existence of a direct relationship between the bacterial contamination of irrigation water and the contamination levels of subsequent biomass such as foliage, soil, and leachate. Contaminated soil and leachate can generate health risks for future generations of crops, especially those with low growing foliage that have direct contact with the ground. There are potential public health risks from using non-disinfected wastewater effluent to irrigate crops. The results show that leafy greens irrigated with treated wastewater effluents could pose health risks to humans, especially considering the bacteria found have a high probability of being resistant to one or more antibiotic. Overall, the reuse of wastewater as irrigation source for crops attracts enormous interest, mainly in water scarce regions, and its successful application will depend on management strategies to guarantee an inexpensive, efficient, and safe water supply.

## Data Availability Statement

The original contributions presented in the study are included in the article/supplementary material; further inquiries can be directed to the corresponding author.

## Author Contributions

Conceptualization and funding acquisition: TG, RK, EM, and CG. Methodology: HS, CP, TG, RK, and CG. Formal analysis: HS, CP, TG, EM, RK, and CG. Resources: TG, RK, and CG. Data curation, original draft preparation, and visualization: HS, CP, and CG. Review and editing: CP, RK, and CG. Supervision: CG. All authors contributed to the article and approved the submitted version.

### Conflict of Interest

The authors declare that the research was conducted in the absence of any commercial or financial relationships that could be construed as a potential conflict of interest.
